# The short-term mortality fluctuation data series, monitoring mortality shocks across time and space

**DOI:** 10.1038/s41597-021-01019-1

**Published:** 2021-09-06

**Authors:** Dmitri A. Jdanov, Ainhoa Alustiza Galarza, Vladimir M. Shkolnikov, Domantas Jasilionis, László Németh, David A. Leon, Carl Boe, Magali Barbieri

**Affiliations:** 1 Max Planck Institute for Demographic Research, Rostock, Germany; 2 Research University Higher School of Economics, Moscow, Russia; 3 Demographic Research Centre, Vytautas Magnus University, Kaunas, Lithuania; 4 London School of Hygiene & Tropical Medicine, London, UK; 5 UiT Arctic University of Norway, Tromsø, Norway; 6 University of California, Berkeley, USA; 7 French Institute for Demographic Studies, Paris, France

**Keywords:** Epidemiology, Sociology, Interdisciplinary studies, Geography

## Abstract

The COVID-19 pandemic has revealed substantial coverage and quality gaps in existing international and national statistical monitoring systems. It is striking that obtaining timely, accurate, and comparable across countries data in order to adequately respond to unexpected epidemiological threats is very challenging. The most robust and reliable approach to quantify the mortality burden due to short-term risk factors is based on estimating weekly excess deaths. This approach is more reliable than monitoring deaths with COVID-19 diagnosis or calculating incidence or fatality rates affected by numerous problems such as testing coverage and comparability of diagnostic approaches. In response to the emerging data challenges, a new data resource on weekly mortality has been established. The Short-term Mortality Fluctuations (STMF, available at www.mortality.org) data series is the first international database providing open-access harmonized, uniform, and fully documented data on weekly all-cause mortality. The STMF online vizualisation tool provides an opportunity to perform a quick assessment of the excess weekly mortality in one or several countries by means of an interactive graphical interface.

## Abstract (summary)

**Table:** 

Measurement(s)	mortality
Technology Type(s)	digital curation
Factor Type(s)	temporal interval • age • country
Sample Characteristic - Organism	Homo sapiens • Severe acute respiratory syndrome-related coronavirus

Machine-accessible metadata file describing the reported data: 10.6084/m9.figshare.15135684

## Background & Summary

Effective public health responses to epidemics have always required timely and reliable monitoring of the situation. During the last decades, there have been numerous short-term mortality peaks related to influenza, heat waves or winter cold, natural or man-made disasters that have been large enough to show signals at the national level. For example, in recent years seasonal influenza outbreaks in 2014–15, 2016–17, and 2017–18 resulted in substantial mortality elevations in many countries. However, it has taken the disaster of the 2019 SARS-Cov-2 (COVID-19) pandemic to make clear how inadequate existing systems have been for generating rapidly open and comparable international data that are both timely and accurate. Such data are needed for the first-line epidemiological and policy response and for projecting the probable trajectory of epidemic spread. At the beginning of the pandemic, the monitoring of the rapidly changing situation was a major challenge for statistical and public health systems. Information about key parameters of the pandemic such as the incidence of new SARS-CoV-2 cases and deaths from COVID-19 were biased by large differences in approaches to testing SARS-CoV-2 and recoding of COVID-19 as a cause of death^1^. While some countries tend to attribute to COVID-19 all or nearly all deaths of those with positive tests for the virus, others applied more conservative approaches with an emphasis on pre-existing co-morbidities. Unfortunately, this huge disadvantage of published data on confirmed cases and deaths due to epidemic diseases (e.g. COVID-19) is underappreciated in the wider research community.

An alternative approach to measuring the population impact of short-term mortality fluctuations such as that due to COVID-19 is quantifying the all-cause mortality burden based on estimating weekly excess deaths relative to what would be expected based on the experience of previous years. This side-steps the serious methodological problems inherent in trying to account for various biases in case ascertainment or certification of cause of death. Modern national statistical systems that have been established in most high-income countries during the last two decades should in principle be able to generate quickly the necessary counts of deaths by week over a number of years.

**Fig. 1:**
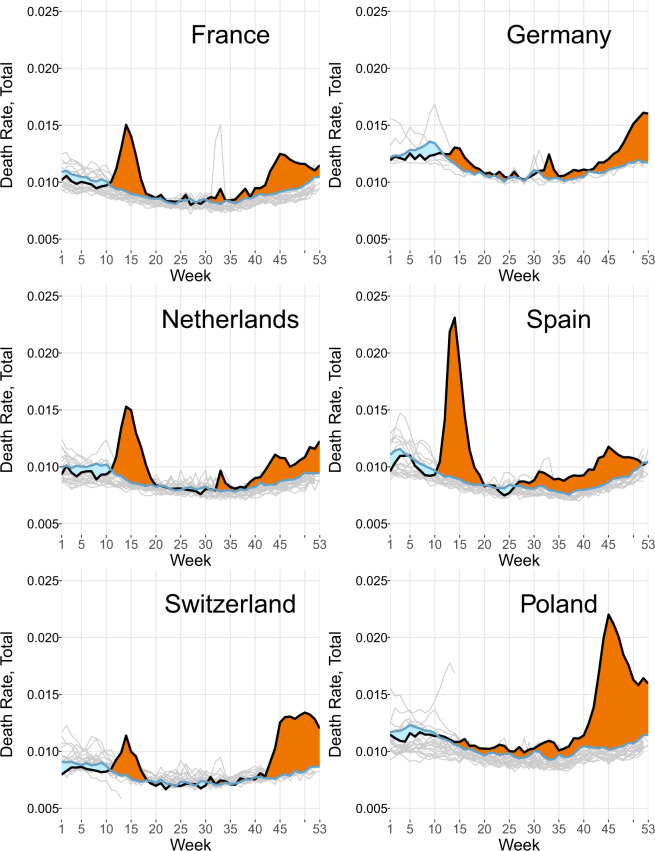
Weekly death rates and excess mortality in six European countries for both sexes and all ages combined. Death rates per person in excess (positive numbers, orange areas on the graph) or deficit (negative numbers, blue areas) mortality are defined by difference between the weekly death rates observed in 2020 and the expected weekly death rates equal to the average weekly death rates over 2015–19 for the same weeks. Note: The figure was constructed using the STMF Visualization Toolkit located at https://mpidr.shinyapps.io/stmortality/.

Figure 1 presents a simple analysis of excess mortality by weeks of 2020 compared to the expected death rates which reflect the average mortality experience of the last five years for the same weeks. In this case, we assumed the average of week-specific values observed during few last years as an expected level of mortality. This is the most commonly used approach. There are numerous methods that might be used to define the reference level^2–4^. The STMF visualization toolkit offers six different options for establishing the reference level. This feature allows to assess the sensitivity of excess mortality estimates on the basis of the selected reference level^3^.

Figure 1 reveals an impressive range of COVID-related mortality patterns in six countries. The first wave of the pandemic generated minor mortality excesses in Poland and Germany and a moderate elevation in Switzerland. Very substantial losses during the first wave in the Netherlands and France and extremely high losses in Spain were followed by moderate losses during the second wave. Poland showed the highest excess mortality during the second wave and Germany and Switzerland had higher excess mortality than during the first wave. Factors and mechanisms underlying these contrasting patterns are still unclear and their scientific investigation should be a priority.

Before the start of the COVID-19 pandemic in March 2020, there were very few countries providing official statistics on weekly death counts and weekly excess mortality. The published data followed a variety of formats which complicated comparisons. Internationally comparable and harmonized data was not available. In response to the global demand for open, reliable, comparable, and timely national (and regional) data, there has been a rapid improvement in the public availability of weekly death statistics since April 2020. Many national statistical offices have started producing and publishing weekly death data in the public domain. Since May 2020, Eurostat has provided a repository for such data^5^. This new and valuable data resource has several limitations: 1) it includes only the European Union countries that voluntarily upload their weekly data; 2) there is very little country-specific information about the data; 3) the data are published “as they are” provided by national statistical institutes without (necessary in some cases) processing or verification.

In May 2020, in response to the COVID-19 pandemic, the Human Mortality Database (HMD)^6^ team established a new open data resource on weekly mortality: the Short-term Mortality Fluctuations (STMF) data series. The present “Data descriptor” describes the STMF.

STMF is the first international scientific database providing harmonized, uniform, and fully documented data on weekly deaths and all-cause mortality. Transparent methodology, rigorous data quality checking procedures, and careful documenting of the original data sources implemented in the STMF strengthen the scientific evidence on the scale of the pandemic and facilitate research on the effectiveness of varying policy responses.

## Methods

The raw data provided by our various sources are checked and processed prior to inclusion in the STMF database. We distribute deaths at an unknown age and split data by age and sex. Nevertheless, the data is not adjusted for death undercount nor smoothed. All known country-specific quality issues are documented in the country-specific metadata file.

STMF covers a subset (38 out of the 48) of countries or areas included in the HMD. Consequently, it is limited by design to populations where death registration and census data are virtually complete, one of the main criteria for inclusion in the HMD. Since a well-functioning vital registration system requires substantial resources, all the STMF countries are developed and relatively wealthy nations.

The length of the country-specific data series depends on the availability of weekly mortality data collected by the National Statistical Offices. Because of the lack of historical series of such data, the STMF covers a relatively short period. The longest data series is available for Finland and it covers the period from 1990 to 2020. Most of the country series (23 out of the 38) begin in 2000. Chile, Greece, and Germany have the shortest data series, beginning in 2016/17 (see details in the “Usage Note” section). The Russian data series does not include data for 2020. The Russian Federal State Statistical Service publishes weekly death counts only as part of the standard yearly statistical report, and therefore the counts arrive after a significant delay: data for 2020 is likely to be published in September 2021.

Data processing starts with the collection of annual population exposures and weekly death counts. We use population exposures from the HMD. For the most recent year(s), when the HMD estimates are not available, they are complemented by the forecasted projections. The Lee-Carter model^7^ is used to extrapolate annual deaths that consequently serve as input to estimate age-specific population exposures and death counts under the assumption of zero migration. The Lee-Carter model is fitted using the available HMD data series starting from 2005. The choice of the reference period in the Lee-Carter model may significantly affect the long-term forecast^7,8^ but has little effect on the STMF estimates such as death rates and ungrouped death counts. We used relatively short reference period in order to 1) be consistent across countries and 2) focus only on the most recent changes in mortality. These two conditions are limited us to period starting from the early 2000s. The sensitivity analyses (not presented here) have shown that choosing any population exposure forecasting reference period between 2000 and 2010 produces less than 0.5% change in death rates. Forecasting was implemented using the R package “demography”^9^. In general, the forecasted data were used for the last 3-4 years, i.e. 2018–2021.

In the next step, the original data are split into standard age groups. This procedure is applied for each year independently using proportions from annual age-specific death counts (observed or forecasted):

$${\widehat{D}}_{y}^{w}\left(x,x+a\right)={D}_{y}^{w}\left(x,x+b\right)\cdot \frac{{D}_{y}\left(x,x+a\right)}{{D}_{y}\left(x,x+b\right)},$$

where ${D}_{y}^{w}\left(x,x+b\right)$ denotes the number of deaths in the age interval [*x, x* + *b*) in week *w* of year *y* in the original data; ${\widehat{D}}_{y}^{w}\left(x,x+a\right)$ is the estimated number of deaths in the age interval [*x, x*+*a*) in week *w* of year *y*; ${D}_{y}\left(x,x+a\right)$ is the number of deaths in age interval [*x, x*+*a*) in year *y*; *a* and *b* are the length of age interval in the output and original data respectively. The hat ($\widehat{}$) designates the estimated values.

A similar procedure is applied when sex-specific data are not available. Sex-specific weekly death counts in each age group are estimated using the observed sex-ratio in the annual total death counts in the same age group:

$${\widehat{D}}_{y}^{w,males}\left(x,x+a\right)={D}_{y}^{w,total}\left(x,x+a\right)\cdot \frac{{D}_{y}^{males}\left(x,x+a\right)}{{D}_{y}^{total}\left(x,x+a\right)}.$$

Finally, the age-specific death rates are calculated as follows:

$${m}_{y}^{w}\left(x,x+a\right)=\frac{{\widehat{D}}_{y}^{w}\left(x,x+a\right)}{{E}_{y}\left(x,x+a\right)/52},$$

where ${E}_{y}\left(x,x+a\right)$ denotes annual population exposures in age interval in year *y*. Thus, these rates are directly comparable with annual death rates.

The STMF does not perform any adjustment for data incompleteness. It is inevitable that information about deaths appears in statistics with a certain delay. Post-mortems (when required), medical certification, civil registration, data processing, tabulation, and checking takes time. For this reason, the last available weeks in 2020 for data series by date of occurrence are incomplete. Besides, all data for the current year for all countries, and (in some cases) for the previous year, should be considered as preliminary.

For the STMF, we collect deaths by date of occurrence. This type of data is preferred for analysis since artificial fluctuations typically affect death counts by date of registration. However, depending on the country, weekly death counts are sometimes provided by date of registration only. There is no way to convert the date of registration into the date of occurrence. All countries included in the STMF except Northern Ireland, Scotland, and England and Wales provide deaths by date of occurrence. These exceptions provide data according to the week of registration. For each country, the exact type of input data is indicated in the metadata file as well as in the input file providing the original raw death counts.

## Data Records

**Fig. 2:**
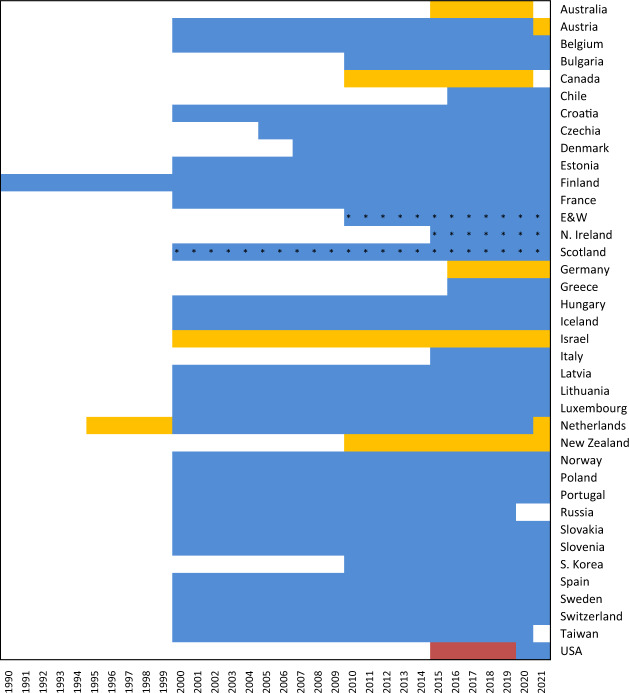
Data availability in the STMF. Colored rectangles indicate availability of weekly-specific data for the corresponding years. Nevertheless, for the first year in the data series, a few first weeks might be missed while, for the last year, data collection might still be in progress and the last weeks might be missed. A blue color indicates that the original data are provided by sex and for age groups detailed enough to create STMF age groups without additional adjustments. A yellow color indicates that at least some of the original data for that year are provided by broad age group and sex. The red squares point to original data available only by broad age groups, i.e. STMF data were split by age and sex. A star (*) indicates that data is available only by date of registration instead of date of occurrence.

The STMF provides open and easy access to death counts and rates for all-cause mortality cross-classified by week, year, sex, age, and country in a standard format along with a complete description of data formats, data processing, and methods. The database contains also the original input data used to produce these output estimates. In addition, detailed country-specific documentation is provided along with precise references to the source and provenance of the original data. Figure 2 provides information about data availability across all STMF countries.

The data, metadata, and detailed documentation are freely available for all users at the HMD website (www.mortality.org). We use CC-BY 4.0 license^10^ for the STMF data. The snapshot of the dataset described here is available at the figshare repository^11^. A detailed description of data formats and methods is given in the STMF Note^12^. The country-specific metadata including information about data sources and shape of the original data is provided as a separate PDF file. We also provide original raw data used for calculation in a standardized format as a set of comma-separated (CSV) files.

STMF data series are presented in tabular form. Data is available in two formats: as an Excel file and as a comma-separated (CSV) file. Both files contain all available data series and provide the same data in two different formats. Each line (record) in the Excel and CSV files has an identical structure. The first four columns of the data matrix define the country (ISO-3 country codes), year, week of occurrence or registration, and sex (males, females, and both sexes combined) for the death counts and death rates that are showed in the next columns. The rates and counts are given by age groups (0–14, 15–64, 65–74, 75–84, and 85+) and for all ages combined. STMF death rates are defined as weekly rates, i.e. number of deaths per person-week (see Methods section). The last three columns include explanatory indicators: “*Split*” indicates whether the data were split from aggregated age groups; “*SplitSex*” indicates if the original data are available by sex (0) or data was split by sex (1); “*Forecast*” is equal to 1 for all years where forecasted population exposures were used to calculate the weekly death rates. The Excel file also provides a summary information relative to the whole dataset on the first worksheet. The following worksheets present country-specific data. Each worksheet contains all of the series available for the corresponding country.

The choice of the age groups was defined by the epidemiological features of the COVID-19 pandemic. The age scale in the original data provided by the National Statistical Institutes varied across countries and periods. Some countries provide data by 5-year age groups while others use extremely broad age intervals (e.g. 0–17, 18–64, 65+). Despite a number of methods for ungrouping aggregated vital statistics^13–16^, none of them was designed for the specific case of weekly death counts with elevated mortality due to pandemics. In order to minimize a possible model-related bias and the associated misinterpretation of the data, we decided to focus only on broad age groups. The age distribution of COVID-19 deaths in age groups below 65 is very consistent across different settings^17^, while excess mortality in 2020 is disproportionally concentrated at the old ages^18,19^. Thus, the standardized output file includes five age groups: child mortality (0–14), adult mortality (15–64), and three age groups for old-age mortality (65–74, 75–84, 85+). Meanwhile, the STMF also provides data in their original classification to allow users to reclassify data using a different age grouping depending on their specific needs.

The raw data provided by our various sources are checked and processed prior to inclusion in the STMF database. We distribute deaths at an unknown age and split data by age and sex. Nevertheless, the data is not adjusted for death undercount and is not smoothed. Raw data is provided as a set of country-specific files. Each record in the data file refers to the death count for one particular week. Each death count (field “*Deaths*”) corresponds to a particular population/country name (field “*PopName*”), geographical area (field “*Area*”), calendar year (field “*Year*”), calendar week (“Week”), sex (field “*Sex*”), age/lower limit of age interval (field “*Age*”), the length of the age interval (field “*AgeInterval*”), the type of data (field “*Type*”), and the type of data access (field “*Access*”).

All known country-specific quality issues are documented in the country-specific metadata dossier with a standardized structure. The metadata systematically includes the following sections: (statistical) coverage, time coverage, (format and shape of the) original data on death, manipulation applied to the original data to obtain the output data, information on the data source and data provider, additional notes, and references (to the data sources). The metadata is presented as a single PDF file.

To facilitate research on mortality outbreaks and seasonal variations by professional users and to enhance basic understanding of the data by non-professional users, we added a visualization layer to the database^3^. The STMF visualization tool is an open-sourced, web-based shiny^20^ application focusing on displaying excess mortality in weekly death counts or death rates. The tool can be accessed at https://mpidr.shinyapps.io/stmortality.

## Technical Validation

The STMF is limited to populations included in the HMD. The selection criteria to include countries in the HMD rely on completeness of coverage and general quality of statistics on deaths, births, and population exposures. Before deciding to include data for a new country, extensive checks are performed to assess the reliability of the input data and the coverage and quality of population statistics of the country^21^. Thus, the STMF includes only countries with virtually or fully complete coverage of vital statistics and meeting strict formal criterions regarding the data quality.

In addition to country-specific knowledge based on the HMD experience, a set of additional data quality indicators is computed to check consistency of the STMF data series during each update. These indicators include both internal (sums by age and sex match respective totals; processed death counts against the raw data; sex ratios in death rates) and external (sum of weekly deaths counts over the year is close to yearly official figures) consistency checks. A series of diagnostic charts are produced to check the plausibility of seasonal trends, consistency of mortality trends over time, and to detect possible outliers. All suspicious cases are carefully investigated by internal experts. If needed, we contact the official data provider to clarify the issue. All unresolved data issues are carefully documented in the metadata.

### Sensitivity to methods

The main goal of the STMF is to provide data for research purposes. The specific aim of the STMF is to provide high-quality consistent data free of any bias caused by methods or models used to adjust the original statistical information. Following this principle, the SMTF puts maximum efforts to collect the most-detailed original data. Unfortunately, the detailed age-specific weekly death data are not always available. For example, eight out of 38 countries provide data only by very broad age groups (Fig. 2). For the period before 2019, the USA publishes the weekly death counts only for both sexes combined.

As described in the methods section, the SMTF methodology foresees four possible adjustments of original death and population exposure counts. Three of these are dealing with the death counts and have to do with the redistribution of death of unknown ages and the splitting of deaths by age and sex. In addition, the SMTF forecasts annual population exposures and death counts for the most recent years.

Summary of potential effects of the aforementioned adjustments:

1. Proportional distribution of deaths of an unknown age. Though commonly used, this approach may lead to an overestimation of infant and old-age mortality. In the STMF, the proportion of deaths at unknown ages is low - below one percent at the very most – and does not have any significant influence on the result.
2. Splitting broad age groups. We apply the age distribution of annual death counts to weekly data. This is a reasonable solution for stable trends. In the case of substantial short-term fluctuations, this assumption can be violated by a shift in the age distribution. For example, COVID-19 deaths are disproportionally concentrated at the old ages^18,19^. In order to minimize the risk of bias, we use only broad age groups.
3. Splitting by sex. The aggregated deaths for both sexes are split using the observed sex proportions in the annual death data. This may lead to sex-specific bias in estimates of excess mortality if the short-term risk factor is sex-specific, e.g. during the war conflicts. Fortunately, for the period of COVID-19 pandemic, all original data are available by sex.
4. Lee-Carter forecast. The forecasted population exposures are used to calculate death rates, whereas annual forecasted death counts are applied to split broad age groups for a few countries (see Fig. 2 for the list of countries and years). The SMTF uses short-term forecasts ranging from one to four years, depending on the availability of annual data. As noted in the Methods section, the output data are not sensitive to the choice of the reference period in the forecasting model. Moreover, as long as variations in the short-term forecast are moderate, our estimates are stable and do not depend on the choice of the forecasting model.

## Usage Notes

Currently, the STMF dataset includes weekly death counts and rates for 38 countries/areas (see section Data Records for details). We plan to include more data series shortly and provide periodic updates based on the most recent data available from each country. In the short term, the project team aims at ensuring timely access to the most recent weekly mortality data for researchers, the media, and policymakers during the COVID-19 pandemic. In the longer-term, the STMF is seen as a continuously updated dataset that will be useful for addressing mortality fluctuations, not only from epidemic diseases but also from other acute episodes with impacts on national mortality rates. Thus, users are encouraged to visit the HMD website (www.mortality.org) to get the most recent version of the dataset.

The STMF collection is a unique data resource aimed to provide reliable and uniform mortality data for scientific investigation.

The main advantages of the STMF are that:

- The database follows four main guiding principles first formulated for the HMD^6,21^:

- °comparability of the series across time and space;
- °use of transparent and standardized methods for processing the data;
- °uniform presentation of the data in XLSX and CSV formats facilitating their use by various groups of potential users; free and easy access to the data, metadata, and documentation;
- °reproducibility via extensive documentation and provision of original input data.

- In addition to weekly death counts, the STMF provides age-specific death rates.
- The data series are regularly updated (currently weekly). In the medium-long term, we expect to update the data series quarterly. However, whenever critical circumstances call for it, as in the current COVID-19 pandemic, the series will be updated according to the required frequency.
- Users are encouraged to contact the team with any concerns or questions regarding the country series in general or specific data points in particular.
- An online visualization toolkit providing easy access and graphical representation of excess mortality estimates is available on the website.
  The STMF has several limitations:
- It provides series at the national level though short-term mortality fluctuations may vary within countries.
- It doesn’t provide data by cause of death. In theory, such statistics might be added to the available data series but would not include the most recent years, because tabulations by causes of death need much more time for processing and therefore, they may be published with very long delays.
- Data quality requirements impose a restriction on the number of populations that can be potentially included in the STMF.

The STMF provides data of high quality in a standardized format. There are, however, a few points that a user needs to be aware of.

1. The data on deaths are classified by date of registration or date of occurrence, depending on the country. There is no way to convert the date of registration into the date of occurrence. The exact type of data and further details on the data collection system relevant for the STMF are indicated in the metadata file. If the data are provided by date of registration (as it is in England and Wales, Northern Ireland, and Scotland), there could be artificial fluctuations in weekly death figures related to various circumstances (e.g. end of statistical periods, weekends, public holidays) as well as moderate shifts in trends and their turning points^22^.
2. Data for the most recent periods are provisional for all countries. Furthermore, data for the last available weeks are incomplete due to the registration lag (time between deaths and time of their registration). The degree of incompleteness varies across countries, time, and age groups. Thus, statistical offices revise these data in the course of future updates. Usually, the weekly data is subject to revision until the yearly final tabulations are officially published (usually in the next calendar year). Each STMF country update takes these revised data into account in addition to any new data point. Each update of the annual data series in the HMD implies the recalculation of the STMF series in order to take into account the most recent HMD data, including population exposures.
3. Deaths and death rates are provided by calendar week starting from Monday and following the general ISO-8601 guidelines. Therefore, all weeks contain 7 days, including the first and the last weeks of the year, which is essential for comparability. There are four exceptions regarding the week beginning day: for the USA, weeks begin on Sunday; for England and Wales and Northern Ireland, on Saturday; and for Australia begins on January 1^st^. Nevertheless, as the also include 7-day weeks, data are comparable. Details are provided in the metadata file.
4. Each year in the STMF refers to 52 weeks. In some cases, the first week of a year may include several days from the previous year and the last week of a year may include days (and their corresponding deaths) from the next year. In particular, it means that a statistical year in the STMF is not exactly equal to the statistical year in the annual country-specific statistics.

## Data Availability

All R scripts used to process the STMF data are available at Zenodo^23^.
